# Optimal timing of surgery for gastric cancer after neoadjuvant chemotherapy: a systematic review and meta-analysis

**DOI:** 10.1186/s12957-023-03251-y

**Published:** 2023-12-01

**Authors:** Qi Ling, Shi-Ting Huang, Tian-Hang Yu, Han-Lin Liu, Lin-Yong Zhao, Xiao-Long Chen, Kai Liu, Xin-Zu Chen, Kun Yang, Jian-Kun Hu, Wei-Han Zhang

**Affiliations:** 1 Department of General Surgery, West China Hospital, Sichuan University, Chengdu, 610041 Sichuan Province China; 2 Gastric Cancer Center, West China Hospital, Sichuan University, Chengdu, China; 3 West China School of Medicine, Sichuan University, Chengdu, China

**Keywords:** Neoadjuvant chemotherapy, Advanced gastric cancer, Overall survival, Disease-free survival, Time to surgery

## Abstract

**Background:**

Following neoadjuvant chemotherapy, surgical resection is one of the most preferred treatment options for locally advanced gastric cancer patients. However, the optimal time interval between chemotherapy and surgery is unclear. This review aimed to identify the optimal time interval between neoadjuvant chemotherapy and surgery for advanced gastric cancer.

**Methods:**

Beginning on November 12, 2022, we searched the PubMed, Cochrane Library, Web of Science databases, and Embase.com databases for relevant English-language research. Two authors independently screened the studies, assessed their quality, extracted the data, and analyzed the results. The primary goal was to investigate the relationship between the time interval to surgery (TTS) and long-term survival outcomes for patients. This study has been registered with PROSPERO (CRD42022365196).

**Results:**

After an initial search of 4880 articles, the meta-analysis review ultimately included only five retrospective studies. Ultimately, this meta-analysis included 1171 patients, of which 411 patients had TTS of < 4 weeks, 507 patients had TTS of 4–6 weeks, and 253 patients had TTS of > 6 weeks. In survival analysis, patients with TTS of > 6 weeks had poorer overall survival outcomes than patients with TTS of 4–6 weeks (*HR* = 1.34, 95% *CI*: 1.03–1.75, *P* = 0.03). No significant differences were found in terms of disease-free survival the groups.

**Conclusion:**

Based on the current clinical evidence, patients with locally advanced gastric cancer may benefit better with a TTS of 4–6 weeks; however, this option still needs additional study.

## Introduction

As the fourth greatest cause of cancer death worldwide and the fifth most common malignancy overall, stomach cancer affects approximately a million people annually and is a primary source of cancer diagnosis worldwide [1]. The prognosis of gastric cancer patients varies with different tumor stages. Advanced tumor stage was the main reason for the tumor burden for gastric cancer patients [2, 3]. In China, more than 70% of gastric cancer patients are in advanced stages once the diagnosis is confirmed [4, 5]. Therefore, there has been a considerable emphasis over the past several decades on discovering methods to increase the chance of survival for patients with locally advanced gastric cancer (LAGC). Since the MAGIC trial was published more than 10 years ago, neoadjuvant chemotherapy has been extensively discussed as part of the comprehensive treatment for LAGC [6]. Neoadjuvant chemotherapy (NCT) has been confirmed in subsequent studies to decrease tumor stage, eliminate micrometastasis, improve tolerance, increase the possibility of radical resection, boost the resection rate of patients with R0, and eventually increase patients’ overall survival times [7–10]. For locally advanced gastric cancer, neoadjuvant chemotherapy is recommended according to the gastric cancer guidelines of the National Comprehensive Cancer Network (NCCN) [11].

In the implementation of neoadjuvant chemotherapy, there are many clinical details that need attention during clinical practice, such as individual chemotherapy drug selection, selection of preoperative cycles, and interval time to surgery when finishing the preoperative chemotherapy. The interval time to surgery is a problem that needs to be comprehensively considered and evaluated by physicians and surgeons for gastric cancers. Patients whose symptoms have not yet resolved due to chemotherapy-related toxicities, deterioration of nutritional status, or serious comorbidities may have worse outcomes following surgery [12, 13]. However, concomitant surgical delay may worsen the prognosis, cause emotional suffering, and lower the quality of life [14, 15]. There is currently no consensus on the appropriate interval time to surgery (TTS) following the completion of NCT for LAGC, although primary tumor excision is often conducted within a few weeks following the last preoperative chemotherapy dose. Augustinas et al. [16] suggest that a greater rate of major pathologic response (mPR) was seen when there was less than 30 days between the conclusion of NCT and gastrectomy, while Liu et al. [17] described an interval time of more than 6 weeks as having relatively high odds of pathologic complete response (pCR).

Therefore, we conducted this research to analyze the optimal time interval from the end of chemotherapy to surgery for LAGC. The overall survival outcomes between different time intervals (< 4 weeks, 4–6 weeks, and > 6 weeks) were the primary endpoints in the present study.

## Materials and methods

The Preferred Reporting Items for Systematic Review and Meta-Analysis Protocols (PRISMA-P) 2015 [18] criteria were followed throughout the course of this systematic review and meta-analysis. On October 18, 2022, we prospectively registered this study on PROSPERO with the identifier CRD42022365196 as part of our project.

### Literature search

To find relevant studies, a systemic search was performed on the PubMed, Cochrane Library, Web of Science databases, and Embase.com, with some adjustments made to the subject words and free words to make it more specific to each database. The search strategies were developed by QL, and the PubMed search strategies were as follows: ((“Stomach Neoplasms”[Mesh]) OR (((((Gastric Neoplasms[Title/Abstract]) OR (Stomach Cancers[Title/Abstract])) OR (Gastric Cancer[Title/Abstract])) OR (gastric carcinoma[Title/Abstract])) OR (gastric tumor [Title/Abstract]))) AND (("Neoadjuvant Therapy"[Mesh]) OR ((((((neoadjuvant chemotherapy [Title/Abstract]) OR (new adjuvant chemotherapy[Title/Abstract])) OR (new auxiliary chemotherapy[Title/Abstract])) OR (preoperative adjuvant chemotherapy [Title/Abstract])) OR (neoadjuvant chemical therapy[Title/Abstract])) OR (new supplementary chemotherapy[Title/Abstract]))). By manually searching the references of the included publications, studies were found that had been missed during the initial literature search. The last date of the search was November 12, 2022.

### Inclusion and exclusion

For consideration in this systematic review, studies were required to fulfil the following conditions: (1) patients have not developed distant metastasis, (2) patients are treated surgically after preoperative chemotherapy, and (3) TTS is documented. The exclusion criteria were as follows: (1) studies that were not reviewed by experts in the field, (2) the relevant information about the patient was not recorded, and (3) languages other than English.

### Literature screening

After the initial search was completed and duplicates were automatically removed, the two authors (Q. L. and S. T. H.) independently reviewed all the articles. First, titles and abstracts were used to sort the papers into relevant groups for further screening. Next, the two authors downloaded the full articles of relevant studies and manually screened them for inclusion and exclusion according to the current study’s inclusion and exclusion criteria. If there were any disagreements, the team talked discussed, and the third reviewers (J. K. H. and W. H. Z.) verified the consensus. Every author was responsible for overseeing the project.

### Data extraction

First, we set up a table to extract the basic information of the studies, and two authors used the same table to collect the data independently. The following data were collected: author, publication year, title, country, type of study, time-to-surgery interval, sample size, stage of patients, regimen of NCT, time of follow-up, perioperative complications, and postoperative survival outcomes. The 3-year OS and DFS data with time-outcome events were extracted from the survival curve. If the necessary information was missing from the primary source, we reached out to the study’s corresponding author to collect this material. Any discrepancies were settled by the third reviewers (J. K. H. and W. H. Z.).

### Quality assessment

Two authors (Q. L., S. T. H.) independently analyzed the five included retrospective studies for methodological quality and resolved disagreements through consultation. Studies were assessed for their quality using the Newcastle–Ottawa Quality Assessment Scale (NOS) [19]. The NOS were classified into three groups based on their characteristics: selection, comparability, and exposure/outcome and then sorted into eight distinct categories. Each high-quality selection and exposure/outcome could receive up to one more star, and the comparability categorization could receive up to two more stars. Finally, according to the stars, the studies were ranked as high (six to nine stars) or low (zero to five stars) quality.

### Statistical analysis

The *I*^2^ and Q statistics were used to assess the degree of heterogeneity between the included studies. Considering the heterogeneity in study characteristics such as patient inclusion and exclusion criteria, time-to-surgery interval heterogeneity, chemotherapy regimens, and medical conditions, *I*^2^ > 50% or *P* < 0.1 was considered substantial heterogeneity. When there was no substantial heterogeneity, the fixed-effects model was selected over the random-effects model. In addition, the random-effects model was utilized. Hazard ratios for survival outcomes were calculated as 95% confidence intervals (CIs). Both fixed and random models were used to examine the possible impact of model choice on the meta-analysis results. The ancillary tools used in this meta-analysis include RevMan 5.4.1 software (RevMan Cochrane Training), Engauge Digitizer software (http://digitizer.sourceforge.net), and R statistical software (Version 4.2.2). In addition, statistical significance is assumed at the *P* < 0.05 level.

## Results

### Study selection

**Fig. 1:**
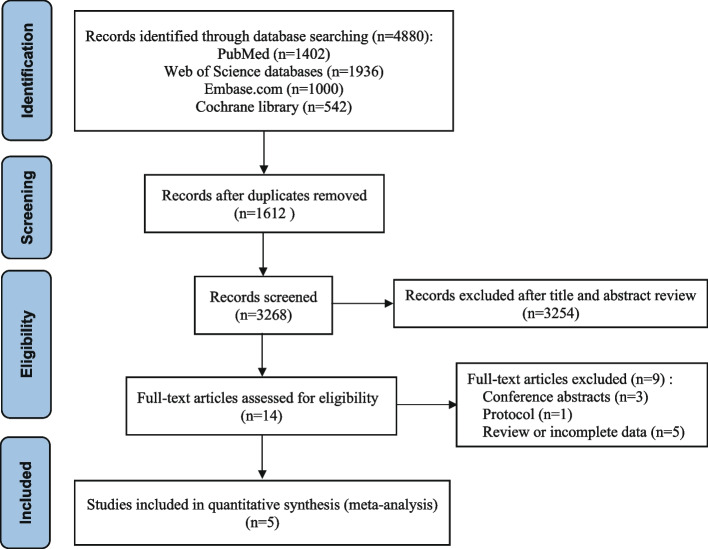
PRISMA selection flow diagram

The number of potentially relevant studies identified through the literature search across four databases was 4880, and after automatic deduplication, 3268 articles were identified as the only literature. After reading the titles and abstracts of the 3268 articles, 3254 were ruled ineligible, leaving 14 articles to be reviewed in full text. Five studies were disqualified because of a literature review or insufficient data; a lack of data led to the exclusion of one protocol study, and three studies were abstracts from conferences. Manually searching the reference lists did not yield any additional studies. There were only five studies [16, 17, 20–22] that satisfied all of the inclusion criteria set by this meta-analysis, so only those results were used. The entire systematic literature review is shown in Fig. 1.

### Study characteristics

Table 1 provides detailed information on the five studies included in this study. These five studies [16, 20–23], published between 2018 and 2021, included 1171 patients with LAGC who were treated with surgery after NCT from 2006 to October 2018. The NCT regimens included S-1 and oxaliplatin (SOX); epirubicin, oxaliplatin, and capecitabine (EOX); capecitabine and oxaliplatin (XELOX); epirubicin, cisplatin, and 5-FU (ECF); docetaxel, oxaliplatin, leucovorin, and 5-fluoracil (FLOT); fluoropyrimidine- and platinum-based doublet (FP); epirubicin, cisplatin, and capecitabine (ECX); and other chemotherapy regimens. The five studies differed in their TTS groupings. Three studies [17, 20, 22] examined three TTS, including > 6 weeks, 4–6 weeks, and < 4 weeks. In one study [16], TTS was divided into ≥ 43 days, 31–42 days, and ≤ 30 days. Patients whose TTS ≥ 43 days, 31–42 days, and ≤ 30 days were classified as belonging to the > 6 weeks, 4–6 weeks, and < 4 weeks, respectively, for the purposes of data analysis. In another study [21], TTS was divided into ≤ 21 days, 22–28 days, 29–35 days, 36–42 days, and 43–84 days. Likewise, TTS ≤ 21 days and 22–28 days combined were considered < 4 weeks, TTS 29–35 days and 36–42 days combined were considered 4–6 weeks, and TTS 43–84 days were considered > 6 weeks. Therefore, the number of patients included in the analysis was 411 with TTS < 4 weeks, 507 with TTS 4–6 weeks, and 253 with TTS > 6 weeks. Based on the NOS assessment [19], five studies were reviewed, with four receiving seven stars (showing high quality) and one receiving eight stars (also indicating high quality).

**Table 1:** Basic characteristics of included studies

Author	Year	Country	Study design	Time intervals and Sample size	stage	NCT	endpoint	NOS^a^
Liu et al.	2018	China	R	<4weeks: 111 4-6weeks: 48 >6weeks: 17	cT2-4, N0-3	SOX; XELOX	pCR; OS; DFS	7
Wu et al.	2019	China	R	<=4weeks: 70 5-6weeks: 103 >6weeks: 56	cT3/4, N+	SOX; XELOX	OS; DFS	7
Juan et al.	2020	Spain	R	<4weeks: 18 4-6weeks: 26 >6weeks: 16	cT2-4, N0-3	ECF; EOX; FLOT	DS; OS	7
Wang et al.	2020	China	R	<=21days: 49 22-28days: 93 29-35days: 108 36-42days:84 42-84days: 92	cT0-4, N0-3	SOX; *XELOX*	pCR; OS; DFS	7
Augustinas et al.	2021	Lithuania	R	<=30days:70 31-43days: 138 >=44days:72	cT2-T4, N+	FLOT; FP; ECX; EOX	mPR; OS; DFS	8

*Abbreviations:* *R* Retrospective study, *SOX* S-1 and oxaliplatin, *XELOX* Capecitabine and oxaliplatin, *ECF* Epirubicin, cisplatin and 5-FU, *Eox* Epirubicin, oxaliplatin and capecitabine, *FLOT* Docetaxel, oxaliplatin, leucovorin and 5-fluoracil, *FP* Fluoropyrimidine and platinum-based doublet, *ECX* Epirubicin, cisplatin, capecitabine

^a^The Newcastle Ottawa Quality Assessment Scale (NOS)

### Overall survival

**Fig. 2:**
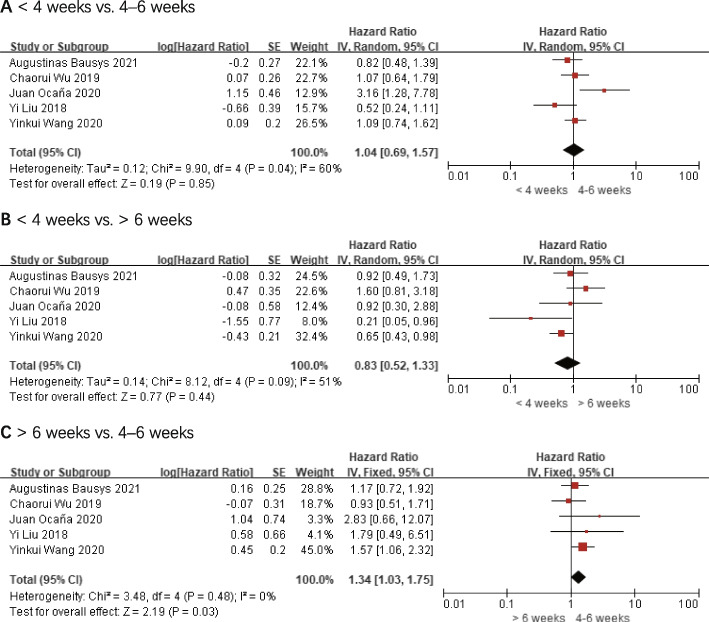
Overall survival (OS). **A** < 4 weeks vs. 4–6 weeks. **B** < 4 weeks vs. > 6 weeks. **C** > 6 weeks vs. 4–6 weeks

The overall survival outcomes were reported in all five studies [16, 17, 20–22]. According to the study’s findings, in terms of overall survival outcomes, no statistically significant differences were found among < 4 weeks and 4–6 weeks (*HR* 1.04, 95% *CI*: 0.69–1.57, and *P* = 0.85) and > 6 weeks (*HR* 0.83, 95% *CI*: 0.52–1.33, and *P* = 0.44). There was a significant decline in overall survival associated with > 6 weeks when compared to 4–6 weeks (*HR* 1.34, 95% *CI*: 1.03–1.75, and *P* = 0.03) and no significant heterogeneity (*I*^2^ = 0%, *P* = 0.48). Figure 2 demonstrates these results.

### Disease-free survival

DFS was noted in four studies [16, 17, 20, 21], and 393 individuals with TTS < 4 weeks, 481 individuals with TTS 4–6 weeks, and 237 individuals with TTS > 6 weeks were ultimately incorporated into the analysis.

**Fig. 3:**
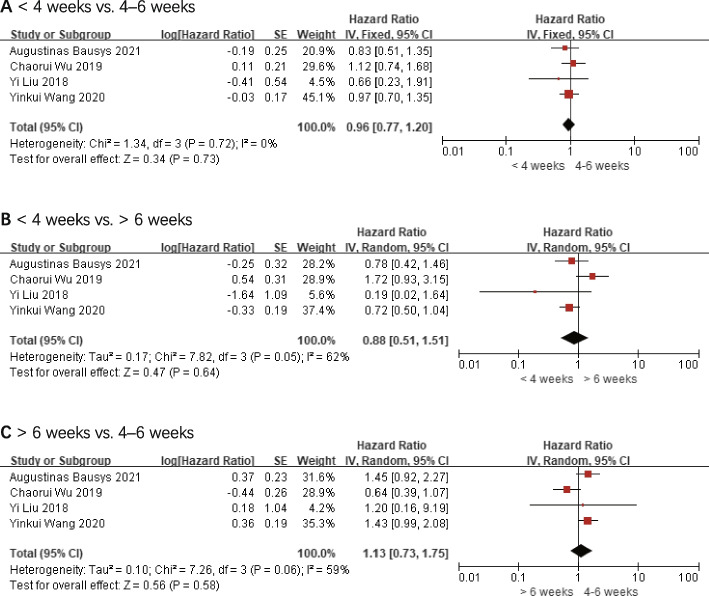
Disease-free survival (DFS). **A** < 4 weeks vs. 4–6 weeks. **B** < 4 weeks vs. > 6 weeks. **C** > 6 weeks vs. 4–6 weeks

The final study revealed that the HR values for DFS were 0.96 (< 4 weeks vs. 4–6 weeks, 95% *CI*: 0.77–1.20 and *P* = 0.73), 0.88 (< 4 weeks vs. > 6 weeks, 95% *CI*: 0.51–1.51, and *P* = 0.64), and 1.13 (> 6 weeks vs. 4–6 weeks, 95% *CI:* 0.73–1.75, and *P* = 0.58), respectively. There were no statistically significant differences between the comparison groups. These outcomes are displayed in Fig. 3.

### Pathological response

**Fig. 4:**
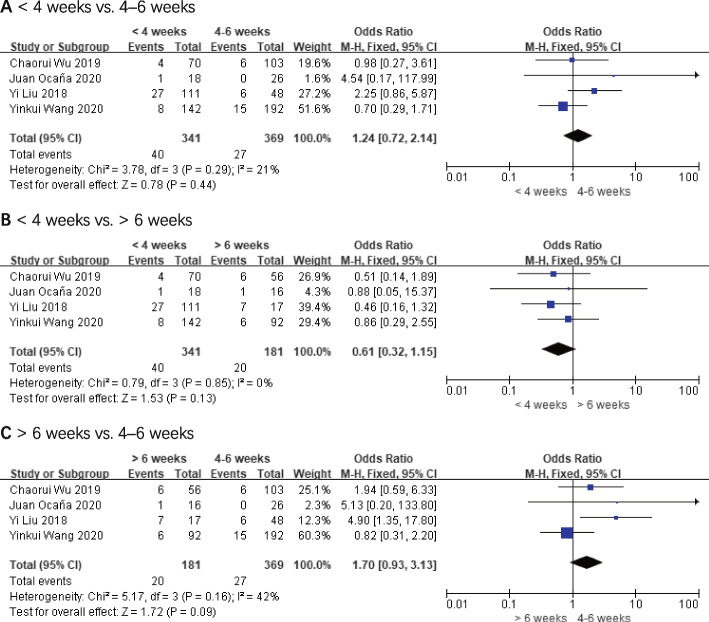
Pathological complete response (pCR). **A** < 4 weeks vs. 4–6 weeks. **B** < 4 weeks vs. > 6 weeks. **C** > 6 weeks vs. 4–6 weeks

Pathological complete response (pCR) data from four of the five studies were published [17, 20–22], and all of the studies included evaluations of curative effects according to RECIST1.1 [24]. According to the results of the final analysis, the odds ratio (OR) values for pCR were 1.24 (< 4 weeks vs. 4–6 weeks, 95% *CI*: 0.72–2.14, and *P* = 0.44), 0.61 (< 4 weeks vs. > 6 weeks, 95% *CI*: 0.32–1.15, and *P* = 0.13), and 1.70 (> 6 weeks vs. 4–6 weeks, 95% *CI*: 0.93–3.31, and *P* = 0.09), respectively. The comparative groups did not differ significantly from one another. These outcomes are shown in Fig. 4.

**Fig. 5:**
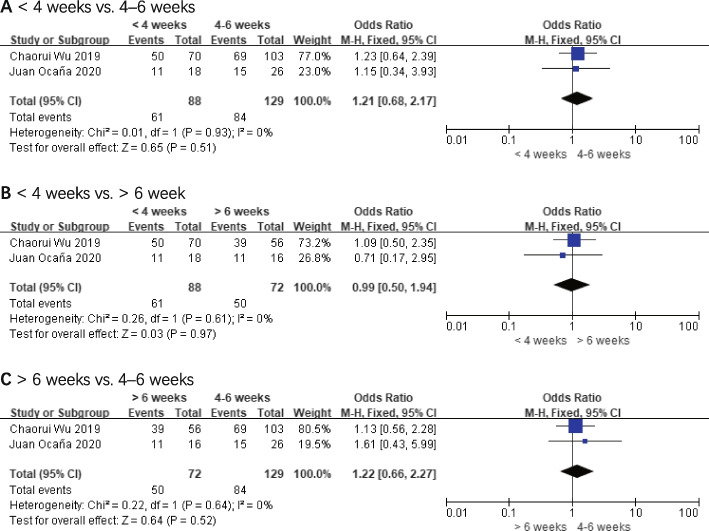
Major pathologic response (mPR). **A** < 4 weeks vs. 4–6 weeks. **B** < 4 weeks vs. > 6 weeks. **C** > 6 weeks vs. 4–6 weeks

Data on the pathologic response were reported in three of the five studies [16, 20, 22]. One of the studies [16] evaluated the curative effect according to Becker et al. [25], which was excluded from the pooled analysis. The other two articles [20, 22] used RECIST1.1 [24], and the analysis results suggested that the OR values for mPR were 1.21 (< 4 weeks vs. 4–6 weeks, 95% *CI*: 0.68–2.17, and *P* = 0.51), 0.99 (< 4 weeks vs. > 6 weeks, 95% *CI*: 0.50–1.94, and *P* = 0.97), and 1.22 (> 6 weeks vs. 4–6 weeks, 95% *CI*: 0.66–2.27, and *P* = 0.52), respectively. The comparative groups did not differ significantly from one another. These outcomes are displayed in Fig. 5.

### Postoperative complications

**Fig. 6:**
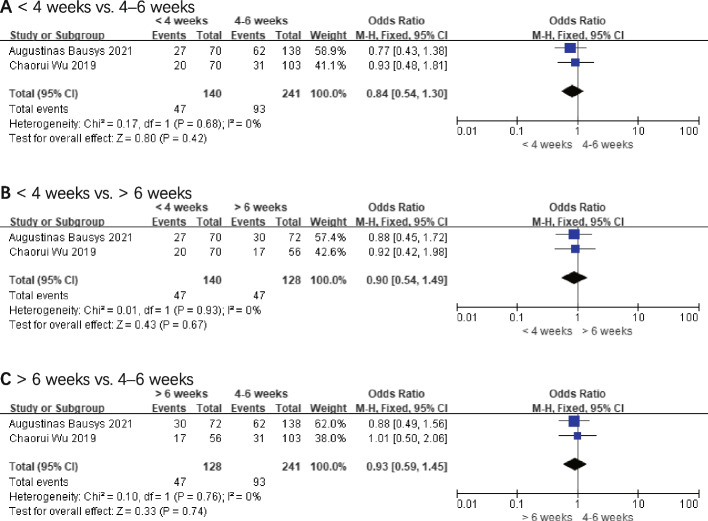
Postoperative complications. **A** < 4 weeks vs. 4–6 weeks. **B** < 4 weeks vs. > 6 weeks. **C** > 6 weeks vs. 4–6 weeks

A total of 36.74% of patients (187/509) experienced postoperative complications according to two papers on postoperative complications [16, 20]. The findings of the final analysis show that OR for postoperative complications was 0.84 (< 4 weeks vs. 4–6 weeks, 95% *CI*: 0.54–1.30, and *P* = 0.42), 0.90 (< 4 weeks vs. > 6 weeks, 95% *CI*: 0.54–1.49, and *P* = 0.67), and 0.93 (> 6 weeks vs. 4–6 weeks, 95% *CI*: 0.59–1.45, and *P* = 0.74), respectively. The comparative groups did not differ significantly from one another. These results are shown in Fig. 6.

## Discussions

This meta-analysis was performed to evaluate whether there is a correlation between the TTS and survival outcomes for patients with LAGC. Although surgery following neoadjuvant chemotherapy at intervals of 4–6 weeks has been adopted in some clinical studies [6, 24, 26], there is no definitive evidence for this interval or for shortening or prolonging it. This research included 1171 individuals with LAGC, and it was concluded that those with TTS 4–6 weeks had improved overall survival; however, there was no appreciable improvement in disease-free survival.

The optimal time to have surgery following neoadjuvant chemotherapy or radiation varies depending on the specialty. Regarding rectal cancer, evidence from several randomized controlled trials has suggested that patients with rectal cancer have higher pCR, better prognosis, and better recurrence-free survival with longer intervals between operations after chemoradiotherapy [27–29]. The results of a meta-analysis of 26 trials showed that the PCR and downstaging rates for patients with rectal cancer were higher, and that there was longer DFS survival without increased surgical morbidity given a delay of at least 8 weeks between the end of NCT and surgery [30]. It is possible that this is because rectal cancer patients’ responses to chemoradiotherapy are time dependent, with complete tumor regression taking several months [31]. These findings suggest that prolonging the surgical interval beyond 8 weeks may facilitate surgical resection rather than impede it. Similarly, in breast cancer research, Rachel A. Sanford et al. [32] sought to delve into the link between TTS and survival outcomes. Their research of 110 breast cancer patients treated with NCT suggested that an interval between NCT and surgery of 4–6 weeks had a favorable effect on OS compared with TTS < 4 weeks or > 6 weeks.

For gastric cancers, many researchers have attempted to find the best TTS to improve the prognosis of patients. Wang et al. [21] categorized 426 patients with LAGC into five groups to determine whether different time intervals to surgery would improve patient outcomes. They found that patients who underwent surgery within 22–35 days had a better OS (*P* = 0.001) and DFS (*P* = 0.017) without increasing postoperative complications or decreasing pCR rates [21]. Liu et al. classified 176 patients into TTS < 4 weeks, 4–6 weeks, and > 6 weeks, indicating that patients with TTS > 6 weeks had a better pCR compared to those with TTS of 4–6 weeks, but OS and DFS were not significantly different [17]. However, Wu et al.’s findings suggest that TTS has no effect on histopathological response or survival results [20]. In our study, we found that patients with TTS of 4–6 weeks have a survival benefit, and those with TTS > 6 weeks or < 4 weeks have no advantage in survival. However, limited by the included research, the number of cases, and the low level of evidence, the optimal TTS selection may be restricted at 4–6 weeks. Integrated current evidence and NCCN guidelines recommend that TTS of 4–6 weeks might be a better option for LAGC patients.

In addition, improving the R0 resection rate and pCR rate is another treatment objective of NCT [33]. The tumor pathological response rate is one of the important evaluation indices of drug treatment. Tumor pathology responses have been demonstrated to be related to increased survival in LAGC, and the major pathological responses have a beneficial effect on OS and pCR [34, 35]. However, in this study, not all studies included reporting of the tumor pathological response rate. Based on the limited number of studies, the TTS and pathological response were not correlated.

Because surgical trauma and gastric cancer lymph node clearing range and neoadjuvant chemotherapy inevitably cause a reaction, tissue edema fibrosis can raise the possibility of complications during and after surgery [36]. The choice of appropriate TTS must also be considered from the perspective of balancing systemic and local reactions and long-term survival after chemotherapy. However, fewer studies have been conducted on the correlation between TTS and intraoperative and postoperative complications; therefore, additional prospective studies are needed to test and confirm these assumptions.

On the other hand, NCT brings a greater survival benefit than chemotherapy after surgery for resectable gastric cancer [37]. A recent meta-analysis reported that TPF (taxane and platinum plus fluoropyrimidine) triple neoadjuvant chemotherapy was more beneficial for pCR, OS, and DFS in patients with gastric cancer than other regimens [38]. NCT may be associated with increased morbidity and mortality [23]. NCT may cause adverse events such as leukopenia, neutropenia, nausea and vomiting, and fatigue in patients, with a higher incidence of triple therapy [38]. Prolonged TTS in some patients may be related to these complications.

### Limitations

The main limitation of the study is that only five retrospective clinical studies were included. The primary aim of this study was to analyze the effects of the time interval to surgery on the outcome of neoadjuvant therapy for gastric cancer patients. The limited number of investigations and the majority of studies are from one country affected the power of the study and could be a of cause research bias. In addition, we did not analyze the effects of different drug regimens of TTS on the efficacy of neoadjuvant therapy. In the present study, we did not limit the neoadjuvant drug regimens that were included in the study. However, all of the studies included in the analysis were not restricted to single chemotherapy regimens but adopted multi-chemotherapy regimens. Therefore, the effects of different drug regimens of TTS on treatment outcome were not analyzed. Last, there are many factors that affect the short-term and long-term efficacy of neoadjuvant therapy. For example, immune status, primary tumor stage, and tumor molecular characteristics are all important influencing factors. Due to the limited number of retrospective clinical studies, the influences of these factors on TTS and treatment outcome were not analyzed in this study.

Although with this, our study suggests that a time interval to surgery of 4–6 weeks after neoadjuvant chemotherapy may be the most appropriate choice for patients with advanced gastric cancer treated with neoadjuvant chemotherapy based on current clinical evidence. Of course, to make better treatment decisions and choose the appropriate TTS for different patients, more high-quality research is worth looking forward to in the future. Perhaps prospective, large sample, and multicenter research will be helpful for solving this problem.

## Conclusions

The current meta-analysis revealed that surgery within 6 weeks after NCT in patients with LAGC is associated with increased overall survival. Certainly, further clinical trials are warranted to ascertain the survival benefit of TTS of 4–6 weeks for patients with gastric cancer.

## Data Availability

On reasonable request, the corresponding author will provide the data that support the conclusions of this study.

## Abbreviations

LAGC: Local advanced gastric cancer
NCT: Neoadjuvant chemotherapy
TTS: Interval time to surgery
mPR: Major pathologic response
pCR: Pathologic complete response
DFS: Disease-free survival
OS: Overall survival
NOS: Newcastle-Ottawa Quality Assessment Scale
